# Stroke subtype classification by geometrical descriptors of lesion shape

**DOI:** 10.1371/journal.pone.0185063

**Published:** 2017-12-07

**Authors:** Bastian Cheng, Christian Knaack, Nils Daniel Forkert, Renate Schnabel, Christian Gerloff, Götz Thomalla

**Affiliations:** 1 Department of Neurology, University Medical Center Hamburg-Eppendorf, Hamburg, Germany; 2 Department of Radiology, Hotchkiss Brains Institute, Calgary, Canada; 3 Department of General and Interventional Cardiology, German Center for Cardiovascular Research (DZHK) partner site Hamburg/Kiel/Lübeck, University Heart Center Hamburg Eppendorf, Hamburg, Germany; University of Münster, GERMANY

## Abstract

**Background and purpose:**

Inference of etiology from lesion pattern in acute magnetic resonance imaging is valuable for management and prognosis of acute stroke patients. This study aims to assess the value of three-dimensional geometrical lesion-shape descriptors for stroke-subtype classification, specifically regarding stroke of cardioembolic origin.

**Methods:**

Stroke Etiology was classified according to ASCOD in retrospectively selected patients with acute stroke. Lesions were segmented on diffusion-weighed datasets, and descriptors of lesion shape quantified: surface area, sphericity, bounding box volume, and ratio between bounding box and lesion volume. Morphological measures were compared between stroke subtypes classified by ASCOD and between patients with embolic stroke of cardiac and non-cardiac source.

**Results:**

150 patients (mean age 77 years; 95% CI, 65–80 years; median NIHSS 6, range 0–22) were included. Group comparison of lesion shape measures demonstrated that lesions caused by small-vessel disease were smaller and more spherical compared to other stroke subtypes. No significant differences of morphological measures were detected between patients with cardioembolic and non-cardioembolic stroke.

**Conclusion:**

Stroke lesions caused by small vessel disease can be distinguished from other stroke lesions based on distinctive morphological properties. However, within the group of embolic strokes, etiology could not be inferred from the morphology measures studied in our analysis.

## Introduction

Stroke etiology has a substantial impact on prognosis and risk of recurrence [1,2]. Early identification of stroke etiology may influence specific treatment decisions and enables early initiation of effective secondary prevention. In this context, findings from acute stroke magnetic resonance imaging (MRI) are valuable due to the high sensitivity in the detection of stroke lesions by diffusion-weighted MRI (DWI). Stroke lesion characteristics including localization and distribution of lesions may indicate specific etiology, e.g. small vessel disease or embolic stroke [3,4]. Potential stroke mechanisms are, therefore, commonly inferred from acute DWI datasets and may guide further diagnostics and secondary prevention at an early stage of the diagnostic work-up.

Cardiac embolism represents the second most frequent cause of stroke, and atrial fibrillation (AF) remains the most common cause of cardiac embolism accounting for about one fifth to one third of ischemic strokes [5,6]. Identification of a cardioembolic origin is important since oral anticoagulation provides a highly effective, targeted secondary prevention for this subgroup of stroke patients [7]. However, AF remains undetected in the acute phase of stroke in a significant percentage of patients due to its potentially intermittent occurrence. Detection rates increase with duration of cardiac monitoring. However, considerable delay until detection of AF can be observed leaving patients with suboptimal preventive treatment for relevant time periods [8]. Inference of a cardioembolic etiology from stroke lesion patterns during the acute phase of stroke is, therefore, a desirable goal that has been in the aim of several previous studies, albeit yielding mixed results [9–11].

Recent studies have suggested that the analysis of geometrical features of stroke lesions can lead to valuable parameters for predicting the progression of brain tissue damage after ischemic stroke [12,13]. This analysis approach considers the shape of stroke lesions and quantifies features of the three-dimensional lesion morphology not intuitively accessible by visual inspection or simple volume quantification. In this study, we investigated, whether lesion shape descriptors quantifying three-dimensional lesion morphology in stroke MRI within 24h after stroke symptom onset differ between stroke subtypes as defined by the ASCOD classification [14]. We hypothesized that distinctive lesion morphology measures identify specific stroke etiologies. Specifically, we investigated the value of lesion morphology characteristics to detect stroke of cardioembolic origin.

## Methods

### Patients

We retrospectively analyzed data of consecutive stroke patients admitted to our Stroke Unit between January 2012 and May 2013. The inclusion criteria were: (1) diagnosis of acute ischemic stroke, (2) MRI examination during the first 24 hours after stroke including fluid-attenuated inversion recovery (FLAIR) and DWI sequences with images of sufficient quality for analysis, and (3) presence of an acute DWI lesion corresponding to the clinical syndrome. This study has been approved by the local ethics committee (Ärztekammer Hamburg). All research was conducted according to the principles of the Declaration of Helsinki. Access to patient information was available to BC, CK, RS, CG and GT to identify potential stroke etiology based on patient record forms. Imaging data was anonymized previous to analysis. Patient treatment was supervised by CG and GT. None of the remaining authors were the treating physicians.

### Stroke subtype classification

All patients were subjected to comprehensive diagnostic evaluation at the stroke unit according to a clinical pathway routinely performed for all stroke patients at our hospital. This included results from blood tests, electrocardiographic (ECG) recording on admission, >24 hours continuous ECG monitoring at the stroke unit, additional 24-hour Holter ECG, carotid and transcranial duplex sonography, transthoracic echocardiography, and magnetic resonance angiography (MRA) of intracranial arteries. Additional diagnostic tests such as contrast-enhanced MRA of extracranial arteries, digital subtraction angiography, transesophageal echocardiography, or extended blood tests were performed if deemed necessary based on clinical evaluation. Data was collected from individual electronic patient records. In addition, clinical data comprising gender, age, severity of symptoms assessed by the National Institutes of Stroke Scale (NIHSS) on admission was recorded.

Stroke subtype classification was based on the ASCOD (revised ASCO classification) Phenotyping of Ischemic Stroke.[14] The ASCOD classification assesses patients for the presence of five major etiological and mechanism categories: atherothrombosis (A), small-vessel disease (S), cardioembolism (C), other causes (O), and dissection (D). Each category is described as grade 1, for a certain potential cause of the index stroke; 2, if causality is uncertain; 3, if disease is present, but unlikely a direct cause of the index stroke; 0 for absent disease, and 9, for insufficient workup. For meaningful group comparisons between different stroke subtypes, we determined the etiology as undetermined if patients were not graded as 1 or 2 for each subtype or had multiple, concurring grade 1 etiologies.

### Magnetic resonance image acquisition and analysis

At our institution, an acute stroke MRI protocol is routinely performed for all acute stroke patients demonstrating a relevant clinical deficit. All MRI studies were performed on a 1.5-Tesla scanner (Magnetom Avanto, Siemens, Erlangen, Germany) with a standard 12-channel head coil using a standardized stroke imaging protocol. For this study, data from DWI sequences were selected for further analysis. DWI-sequences were configured as single-shot, echo-planar imaging (TR/TE = 4000ms/84ms, slice thickness 4 mm, field of view (FOV) 240 x 240 mm^2^ without diffusion weighting (b = 0 s/mm^2^) and applying diffusion gradients in 3 directions with strong diffusion-weighting (b = 1000 s/mm^2^), which were combined to an average DWI dataset.

All images were analyzed using the in-house software ANTONIA, a multi-purpose analysis tool of stroke lesion analysis. This standardized processing pipeline was described previously [15]. In summary, the software performs image registration, automated segmentation of brain tissue and CSF as well as volumetric quantification of multi-parametric stroke data. In brief, ischemic stroke volumes were calculated from seed-based semi-automatic segmentation of apparent diffusion coefficient (ADC) maps. All lesions were visualized in three-dimensional space and the number of lesion components, i.e. binary sub-lesions not connected to each other, was rated visually by two raters (BC, CK). In addition, a “multiple arterial pattern” was defined including patients with lesions located in at least two of the main arterial territories of the brain (left or right internal carotid artery or posterior circulation territory).

### Lesion shape characteristics

**Fig 1:**
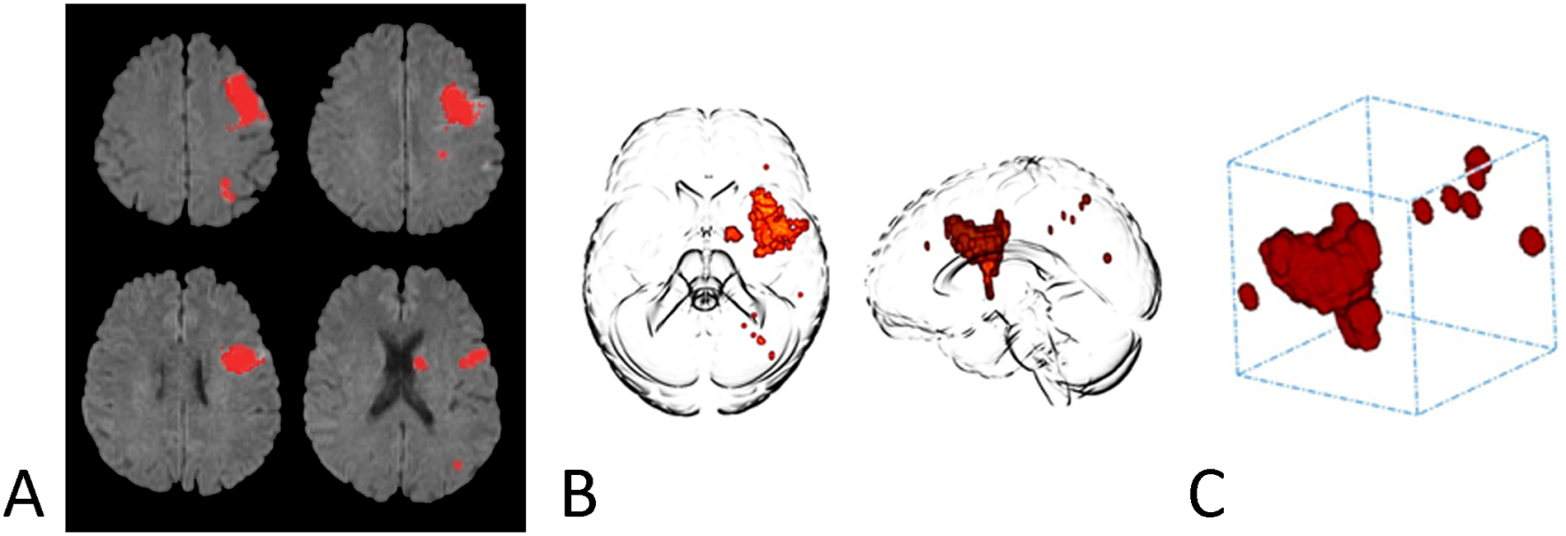
Representative illustration of three-dimensional lesion reconstruction. Stroke caused by arterial dissection of the left internal carotid artery, representations of the acute lesion are shown in red as two-dimensional overlay on diffusion weighted images (A) and as three-dimensional reconstructions (B). Bounding box of the lesion is illustrated in C.

The following measures of lesion morphology were calculated using algorithms implemented in ANTONIA: (1) The surface area (mm^2^) of the stroke lesion; (2) the oriented minimum bounding-box of a lesion (ml): The oriented minimum bounding box is the smallest rectangular volume enclosing all lesion components, whereas the orientation is independent from the coordinate axes; (3) the ratio between bounding-box volume and lesion volume: This value characterizes the amount of volume of the bounding box filled by the actual stroke lesion. Smaller values are observed in more compact lesions, whereas holey, scattered or more ragged lesions demonstrate larger values (Fig 1); (4) the normalized shape factor S of the ischemic lesion, which describes how closely the lesion resembles the shape of a sphere. It is mathematically defined as:

$$S\;=\;(\sqrt{A}/∛V)/2.199085233,$$

whereas A denotes the surface area and V the volume of the lesion. This index gives a value of 1.0 for a sphere and increases the more the shape differs from a perfect sphere.

### Statistical analysis

In a first analysis, group differences between mean values of stroke lesion volume, number of lesion components, surface area, minimum oriented bounding box volume, ratio of minimum oriented bounding box volume and lesion volume, as well as shape factor were compared between ASCOD stroke subtypes as defined above (atherosclerosis, small vessel disease, cardioembolism, other causes, dissection, and undetermined cause). A general linear model was applied using each measure of lesion morphology as dependent variable and stroke subtypes as group. Bonferroni-corrected post-hoc comparisons were carried out to detect significant differences between stroke subgroups.

In a second analysis, we selected only patients with embolic strokes of known etiology, either of a cardiac or non-cardiac source. For this analysis, patients with lacunar infarcts caused by small vessel disease (S1, S2 according to ASCOD) and stroke of unknown origin where excluded. Stroke mechanism of patients previously categorized as C1 and C2 was classified as “cardioembolic”, whereas a “non-cardioembolic” classification was considered in patients previously grouped into A1 or A2 (atherosclerosis), D (arterial dissection) and O (other). Patients classified as “other causes of stroke” (O) were included, since based on history and diagnostic findings, embolic causality was assumed in these cases. We investigated, whether lesion morphology characteristics were able to identify patients with cardioembolic stroke. Therefore, an independent t-test was used for group comparison for each measure of lesion morphology between two groups classified as “cardioembolic” and “non-cardioembolic” strokes as described above. All statistical analyses were performed in SPSS 22.0 (IBM Co., Somers, NY, USA).

## Results

### Patients

Of 189 patients with acute ischemic stroke studied by MRI, 39 patients were excluded due to absence of a lesion in DWI (n = 17) and insufficient image quality (n = 22). In total, 150 patients remained for analysis. Mean age was 77 (95% confidence interval, 65–80) years, 67 (44.7%) were female. The National Institutes of Health Stroke Scale (NIHSS) ranged from 0 to 22 with a median value of 6. Stroke affected the left hemisphere in 70 patients (46.7%), right hemisphere in 56 patients (37.3%) and both hemispheres in 24 patients (16.0%).

### Image analysis and morphological measures

Table 1 summarizes the frequency of stroke etiology for all patients according to ASCOD. We identified atherothrombosis as the underlying etiology in 40 patients (26.7%), small-vessel disease in 33 patients (22.0%), cardioembolism in 37 patients (24.7%), other causes in 10 patients (6.7%), and dissection in four patients (2.7%). Other causes were peri-interventional embolic stroke (e.g. following percutaneous coronary intervention or similar catheter-based procedure) in 9 of 10 cases, and angiitis in the remaining case. Stroke etiology remained undetermined in 26 patients (17.3%).

**Table 1:** Frequency of stroke subtypes for all patients (n = 150) classified with ASCOD phenotyping. Grade 1 applies if a disease was present and potentially causal. Grade 2 is applied if a disease is present but a causal link uncertain. Stoke etiology was regarded undetermined (U) in patients with neither grade 1 nor 2 in any category or presence of multiple grade 1 etiologies.

Stroke subtype	Subtype grade	n	%
Atherothrombosis (A)	A1	18	12.0
	A2	22	14.7
Small-vessel disease (S)	S1	26	17.3
	S2	7	4.7
Cardioembolism (C)	C1	12	8.0
	C2	25	16.7
Other Cause (O)	O1	10	6.7
Dissection (D)	D1	4	2.7
Undetermined (U)		26	17.3

**Fig 2:**
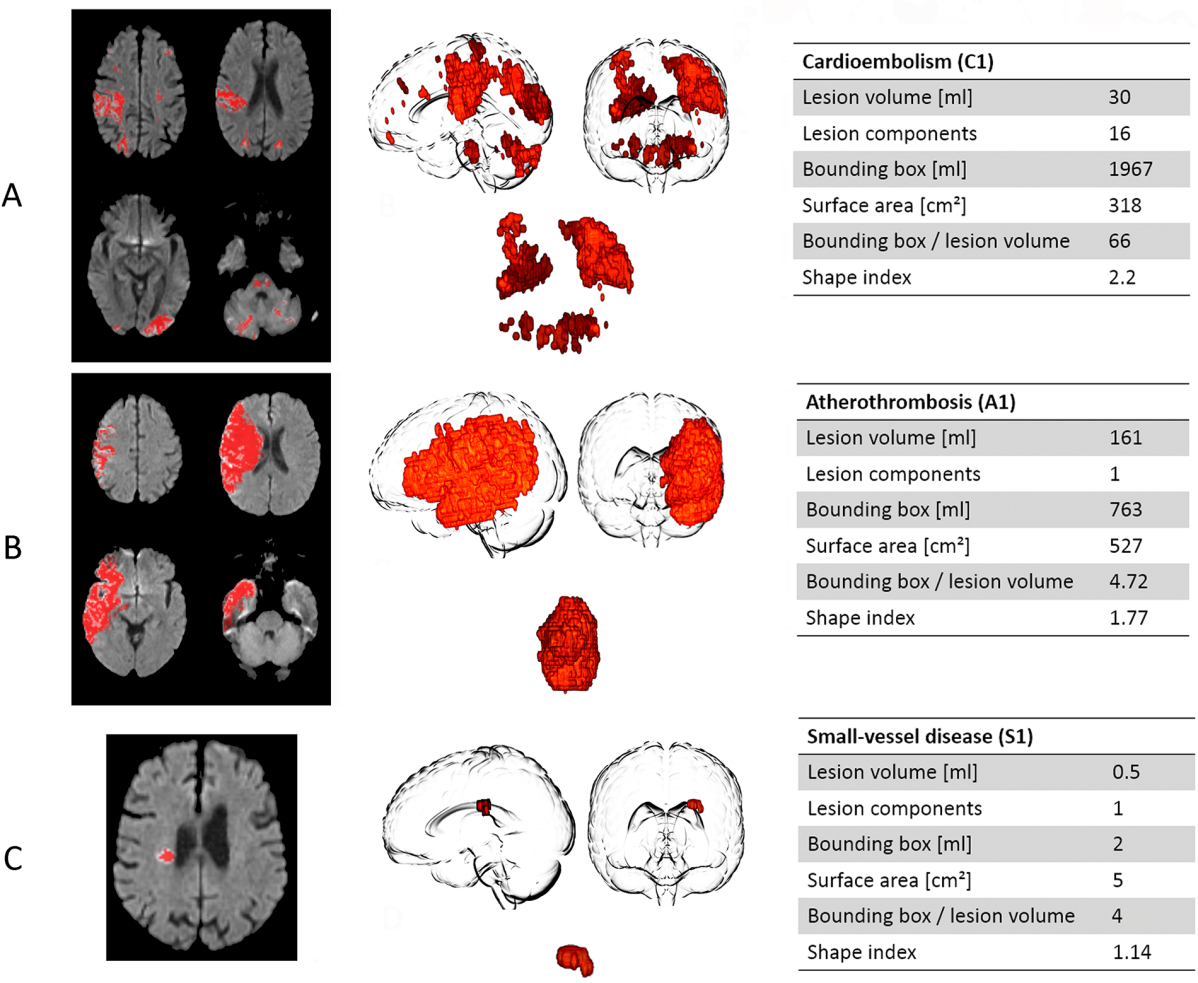
Two- and three-dimensional representations of acute stroke lesions analyzed in this study. Acute stroke lesions are shown in red overlayed on diffusion-weighted images in the first column. Three-dimensional reconstructions are illustrated in the second column. The third column shows application of morphological shape measures for lesions caused by cardioembolism (A), atherothrombosis (B) and small-vessel disease (C).

Fig 2 illustrates three-dimensional reconstructions and morphology measures of different selected stroke subtypes. Mean values of morphological measures and 95% confidence intervals are shown in Table 2. Results from univariate general linear models showed significant effects of stroke subtypes for all measures of stroke lesion shape, specifically lesion volume (F = 13.28; p<0.001), number of lesion components (F = 4.57; p = .001), surface area (F = 15.47; p<0.001), normalized shape factor (F = 19.76; p<0.001), minimum oriented bounding box volume (F = 22.99; p<0.001), and ratio of bounding box and lesion volume (F = 11.04; p<0.001). Post-hoc tests identified significant differences of morphological lesion measures between stroke lesions resulting from small-vessel disease and any other stroke subtypes. Specifically, lesions classified as resulting from small-vessel disease were singular and of smaller size regarding lesion volume, surface, and volume of the minimum oriented bounding box (see Table 2). They also demonstrated significantly lower ratios between the volume of minimum oriented bounding box and lesion volume as well as a lower normalized shape factor indicating a more compact and spherical shape. There were no significant differences regarding the lesion volume, number of lesion components or any other shape measure between the other stroke subtypes. Detailed results of post-hoc tests are given in S1 Table.

**Table 2:** Overview of morphological lesion shape measures grouped by stroke subtype according to ASCOD phenotyping. Number of patients, mean values and 95% confidence intervals are shown. Stoke etiology was regarded undetermined (U) in patients with neither grade 1 nor 2 in any category or presence of multiple grade 1 etiologies.

	Atherothrombosis (A1 and A2)	Small-vessel disease (S1 and S2)	Cardioembolism (C1 and C2)	Other cause (O1)	Dissection (D1)	Undetermined
Number of patients	40	33	37	10	4	26
Stroke volume [ml]	18.6 (7.5–29.7)	0.2 (0.1–0.3)	13.6 (5.2–21.0)	5.5 (0.1–12.4)	21.2 (0.1–79.9)	9.4 (2.7–16.1)
Lesion components	3.9 (2.6–5.2)	1 (1)	3.6 (2.5–4.8)	6.4 (1–11.9)	3 (2–9)	3.4 (2.1–4.6)
Bounding Box [ml]	256.37 (150.6–362.1)	1.4 (0.9–1.9)	228.9 (106.7–351.1)	435.70 (38.5–832.8)	175.01 (0.1–490.7)	173.8 (359.27)
Surface Area [cm^2^]	125.9 (62.2–189.3)	3.5 (2.6–4.4)	99.3 (56.3–142.3)	58.5 (0.7–116.3)	88.2 (1–284.7)	65.8 (32.6–99.1)
Bounding Box / Volume	35.1 (17.2–53.0)	6.5 (5.5–7.5)	41.4 (20.9–61.9)	223.2 (302.60)	16.9 (1.2–52.2)	30.8 (17.4–44.2)
Shape factor	1.7 (1.5–1.8)	1.2 (1.1–1.2)	1.72 (1.3–1.5)	1.74 (1.5–1.9)	1.5 (1.1–1.9)	1.60 (1.5–1.7)

In the second analysis, stroke of potential cardioembolic source was present in 37 patients, whereas a non-cardioembolic etiology was present in 54 patients. In patients with potential cardioembolic stroke, atrial fibrillation was detected in 35 patients, whereas endocarditis was present in two patients. In the second group of patients of non-cardioembolic origin, no atrial fibrillation was detected. Results of univariate group comparisons are shown in Table 3. No significant group differences in lesion volume, number of lesion components, or any measures of lesion morphology were observed between both groups.

**Table 3:** Group comparison of morphological lesion shape measures for patients with embolic strokes of either cardiac or non-cardiac origin. Cardioembolic stroke was defined in patients classified by ASCOD as cardioembolism (C1 and C2; n = 37), a non-cardioembolic origin (n = 54) defined for strokes caused by atherothrombosis (A1, A2), arterial dissection (D1) and other known causes (O1); Multiple arterial pattern is defended as lesions located in at least two of the main arterial territories of the brain (left or right internal carotid artery or posterior circulation territory).

	Cardioembolic stroke (ASCOD C1, C2)	Non-cardioembolic stroke (ASCOD A1, A2, D1, O1)	p-value
Number of patients	37	54	
Patients with AF	35*	0	
Multiple arterial pattern	11	6	
Lesion volume [ml]	13.6 (5.2–21.0)	16.35 (7.7–25.0)	0.66
Lesion components	3.6 (2.5–4.8)	3.1 (2.2–4.5)	0.47
Surface area [cm^2^]	99.30 (56.3–142.3)	110.7 (62.2–159.2)	0.74
Bounding Box [ml]	228.9 (106.7–351.1)	283.6 (181.1–385.0)	0.49
Ratio BB/Volume	41.4 (20.9–61.9)	66.61 (26.8–110.4)	0.31
Shape factor	1.72 (1.3–1.5)	1.65 (1.6–1.7)	0.31

Abbreviations: AF: Atrial fibrillation;

*endocarditis was diagnosed in the remaining 2 patients.

On visual inspection, a multiple arterial pattern defined as lesions located in at least two of the main arterial territories of the brain (left or right internal carotid artery or posterior circulation territory) was present in 11 patients with potential cardioembolic stoke and 6 patients with non-cardioembolic stroke (p = 0.03; Chi-Square test). The sensitivity of a multiple arterial pattern to identify patients with potential cardioembolic stroke was 29.7% (95% CI: 15.9%–46.9%) and specificity 88.9% (95% CI: 77.4%–95.8%). The positive predictive value was 64.7% (95% CI: 42.6%–81.8%), the negative predictive value 64.6% (95% CI: 59.5%–69.9%)

## Discussion

Analysis of three-dimensional stroke lesion shape descriptors revealed characteristic differences between strokes caused by small vessel disease and those of other etiologies. However, no specific differences were found between other stroke subtypes. Specifically, we were not able to identify patients with potential cardioembolic stroke based on morphological measures of acute stroke lesions.

Morphological measures as applied in our study quantify three-dimensional geometric properties of acute stroke lesions that capture complex features of lesion morphology beyond characteristics visible in two-dimensional image analysis. Automatic quantification of these features extracts information from imaging data that is not accessible by qualitative visual rating [16–19]. Analysis of three-dimensional stroke lesion morphology has been used to characterize disease progression in small vessel disease [17] or lesion development in acute ischemic stroke [12]. However, to our knowledge, we provide the first study of lesion morphology as potential indicator of stroke etiology. Lesions caused by small-vessel disease demonstrated distinctive shape characteristics that were not observed in any other stroke subtype. This result comes expected, since lacunar stroke lesions resulting from small vessel disease usually affect single small penetrating brain vessels and share characteristics features such as small volume and singular appearance that can already be appreciated on visual inspection [20]. Indeed, the ASCOD classification defines a potential causality of small vessel disease as a combination of small deep infarcts < 15 mm and other factors such as severe leukoaraiosis or older lacunar infarcts in other territories, among others. Beyond findings also captured by visual inspection, morphological analysis formally described a typical, more compact shape of lacunar lesions by a low ratio between the ratio of bounding box and lesion volume. In addition, a lower normalized shape factor points to the fact that lesions caused by small vessel disease exhibit a significantly different shape that is more similar to a sphere compared to lesions caused by other stroke etiologies.

In view of the well described and homogeneous etiology of lacunar lesions caused by small vessel disease, these results appear to be plausible and suggest that application of morphological descriptors to acute stroke lesions is feasible. However, we were unable to distinguish lesions of any other stroke subtype classified by ASCOD using quantitative shape measurements.

Most non-lacunar ischemic strokes result from emboli either from a cardiac or arterial origin requiring different antithrombotic therapy regimens [21]. Inference of the embolic source from stroke imaging data is, therefore, of value in guiding diagnostic work-up and management of stroke patients. Thus, we further analyzed a group of patients with known embolic strokes by excluding patients with lacunar stroke caused by small vessel disease. In the group of patients with cardioembolic stroke, atrial fibrillation was detected in almost all patients. Atherothrombosis, arterial dissection, and other specific causes were present in the second group of non-cardioembolic stroke (see Table 3). We observed a large variety of lesion patterns and configurations resulting in more or less similar geometrical measurements for both groups. Thus, we conclude that the etiology of stroke lesions considered to be embolic (i.e. non-lacunar) cannot be inferred from the morphology measures studied in our analysis.

Cardioembolic stroke lesions were of similar size as non-cardioembolic stroke lesions, which contrasts previous studies demonstrating that ischemic strokes related to atrial fibrillation tend to be larger on computed tomography imaging in albeit larger populations of stroke patients [22]. This disagreement is most likely caused by the exclusion of macroangiopathic, lacunar strokes in our study. Furthermore, we did not find any difference in number of lesion components between both groups, a finding that reflects the conflicting evidence regarding the association between number of stroke lesions and cardioembolic stroke etiology. Multiple lesions detected by diffusion-weighted imaging or the number of acute and chronic have been associated with atrial fibrillation in previous studies [23,24]. On the other hand, no such finding was detected in 221 patients with atrial fibrillation diagnosed by implantable cardiac monitor during the CRYptogenic STroke And underLying Atrial Fibrillation (CRYSTAL AF) trial [11]. In addition to the amount of ischemic lesions, a multiple infarct pattern involving more than one vascular territory is typically regarded as a marker of cardioembolism, whereas multiple lesions in one vascular territory were linked to atherosclerosis of large cerebral arteries [3]. However, a scattered, multi-vascular lesion pattern was only detected in 13% of 388 patients with a high likelihood of cardioembolic stroke classified by TOAST [25], whereas the majority were classified as large, single lesions restricted to one vascular territory.

In this study, we extended the assessment of lesion distribution beyond counting individual lesions by topologically quantifying lesion distributions. The minimum oriented bounding box of a lesion potentially consisting of multiple components is defined as the smallest rectangular volume enclosing all lesion components (Fig 1). Stroke lesions involving multiple vascular territories would, therefore, result in large bounding box volumes. In addition, multiple, small and scattered lesions would lead to a very high ratio between the volume of the minimum oriented bounding box and lesion volume, whereas this ratio would be smaller for a single lesion of identical volume as illustrated in Fig 2. The ratio between bounding box and lesion volume also quantifies the more or less holey structure of a lesion and has previously been shown to be of value in a model predicting final infarct volumes [12]. In our group of patients, 11 of 37 patients with a potential cardioembolic etiology displayed lesions located in at least two of the main arterial territories of the brain (left or right internal carotid artery or posterior circulation territory), whereas this pattern was only found in 6 of 54 non-cardioembolic stroke patients. Overall, the sensitivity of a multiple arterial pattern to identify a potential cardioembolic stroke etiology was low (30%), which motivates a novel, morphology-based approach. However, neither the volume of the minimum oriented bounding box nor the ratio between bounding box and stroke volume were significantly different between patients with cardioembolic and non-cardioembolic stroke. This finding most likely can be explained by the heterogeneity of lesion configurations and patterns observed in both groups. Indeed, previous studies have found that multiple and various etiologies were implicated with infarcts in multiple cerebral circulations on initial diffusion-weighted MR imaging, including cardioembolic diseases, but also hematologic disorders, angiitis or bilateral or unilateral large-artery occlusions [26]. On the other hand, large single or lacunar lesions have been observed in a relevant proportion of patients with cardioembolism [22,25,27]. Studies also indicate that multiple factors such as platelet function and inflammatory processes influence the pattern of ischemic lesions in cardioembolic stroke via different compositions of cardiac thrombi [25,28].

In the sample analyzed in our study, we observed a representative and characteristic distribution of stroke subtypes [29–31]. A potential stroke etiology was found for the majority of patients due to the availability of data from diagnostic work-up. However, the relatively small sample size restricts the generalizability of our results as a limiting factor. In addition, imaging protocols applied in our study were designed to minimize examination time of acute stroke patients leading to a limited spatial resolution, which might bias the geometric analysis to some extent. Thus, further studies are warranted to evaluate the influence of image quality such as resolution and artefacts on descriptors of lesion shape.

Taken together, our findings support the clinical observation that stroke subtype classification based on visual rating of ischemic lesion pattern is associated with a substantial degree of uncertainty. In this pilot study, we selected basic lesion shape descriptors that are intuitively comprehensible and computationally little demanding. Applying these basic parameters, we were unable to distinguish between non-microangiopathic strokes of various etiologies. However, it is possible that a more detailed analysis involving a higher number of comprehensive features such as signal intensity or lesion texture might lead to different results. Furthermore, advanced machine learning algorithms could be applied to these datasets to discriminate between different stroke subtypes. This quantitative “radiomics” approach has recently been introduced for the classification of tumor phenotypes with promising results [32] and motivates future analogous imaging studies in stroke patients.

### Limitations

As it is true for any diagnostic test (such as visual interpretation of lesion patterns), we would not expect geometrical lesion shape descriptors to be predictive of stroke etiology with unequivocal accuracy.

## Supporting information

S1 Table Clinical and imaging data. Clinical and imaging data including values of geometrical lesion shape descriptors grouped by stroke etiology for all patients included in the study.
(XLSX)

## References

[1] Adams HP, Biller J. Classification of Subtypes of Ischemic Stroke: History of the Trial of Org 10 172 in Acute Stroke Treatment Classification. Stroke. 2015; 1–5. doi: 10.1161/STROKEAHA.114.007773 25813192 10.1161/STROKEAHA.114.007773

[2] Bamford J, Sandercock P, Dennis M, Warlow C, Burn J. Classification and natural history of clinically identifiable subtypes of cerebral infarction. Lancet. 1991;337: 1521–1526. doi: 10.1016/0140-6736(91)93206-O 1675378 10.1016/0140-6736(91)93206-o

[3] Wessels T, Wessels C, Ellsiepen A, Reuter I, Trittmacher S, Stolz E. Contribution of Diffusion-Weighted Imaging in. 2006; PMC7976056 16418352

[4] Kang D-W, Chalela JA, Ezzeddine MA, Warach S. Association of ischemic lesion patterns on early diffusion-weighted imaging with TOAST stroke subtypes. Arch Neurol. 2003;60: 1730–4. doi: 10.1001/archneur.60.12.1730 14676047 10.1001/archneur.60.12.1730

[5] Perera KS, Vanassche T, Bosch J, Swaminathan B, Mundl H, Giruparajah M, et al Global Survey of the Frequency of Atrial Fibrillation-Associated Stroke: Embolic Stroke of Undetermined Source Global Registry. Stroke. 2016;47: 2197–2202. doi: 10.1161/STROKEAHA.116.013378 27507860 10.1161/STROKEAHA.116.013378

[6] Arboix A, Alio J. Acute cardioembolic cerebral infarction: answers to clinical questions. Curr Cardiol Rev. 2012;8: 54–67. doi: 10.2174/157340312801215791 22845816 10.2174/157340312801215791 PMC3394108

[7] Hart RG, Pearce LA, Aguilar MI. Meta-analysis: Antithrombotic Therapy to Prevent Stroke in Patients Who Have Nonvalvular Atrial Fibrillation. Annals of Internal Medicine Review. 2014; 10.7326/0003-4819-146-12-200706190-00007 17577005

[8] Kishore A, Vail A, Majid A, Dawson J, Lees KR, Tyrrell PJ, et al Detection of atrial fibrillation after ischemic stroke or transient ischemic attack: A systematic review and meta-analysis. Stroke. 2014;45: 520–526. doi: 10.1161/STROKEAHA.113.003433 24385275 10.1161/STROKEAHA.113.003433

[9] Novotny V, Thomassen L, Acute NH. Acute cerebral infarcts in multiple arterial territories associated with cardioembolism. 2016; 1–6. doi: 10.1111/ane.12606 27109593 10.1111/ane.12606

[10] Wessels T, Röttger C, Jauss M, Kaps M, Traupe H, Stol E. Identification of embolic stroke patterns by diffusion-weighted MRI in clinically defined lacunar stroke syndromes. Stroke. 2005;36: 757–761. doi: 10.1161/01.STR.0000158908.48022.d7 15746460 10.1161/01.STR.0000158908.48022.d7

[11] Bernstein RA, Di Lazzaro V, Rymer MM, Passman RS, Brachmann J, Morillo CA, et al Infarct Topography and Detection of Atrial Fibrillation in Cryptogenic Stroke: Results from CRYSTAL AF. Cerebrovasc Dis. 2015;40: 91–96. doi: 10.1159/000437018 26182860 10.1159/000437018

[12] Frindel C, Rouanet, Giacalone, Cho T-H, Ostergaard L, Fiehler J, et al Validity of Shape as a Predictive Biomarker of Final Infarct Volume in Acute Ischemic Stroke. Stroke. 2015;46: 976–981. doi: 10.1161/STROKEAHA.114.008046 25744520 10.1161/STROKEAHA.114.008046

[13] Ogata T, Nagakane Y, Christensen S, Ma H, Campbell BC V, Churilov L, et al A topographic study of the evolution of the MR DWI/PWI mismatch pattern and its clinical impact: a study by the EPITHET and DEFUSE Investigators. Stroke. 2011;42: 1596–601. doi: 10.1161/STROKEAHA.110.609016 21512174 10.1161/STROKEAHA.110.609016

[14] Amarenco P, Bogousslavsky J, Caplan LR, Donnan G a., Wolf ME, Hennerici MG. The ASCOD phenotyping of ischemic stroke (updated ASCO phenotyping). Cerebrovasc Dis. 2013;36: 1–5. doi: 10.1159/000352050 23899749 10.1159/000352050

[15] Forkert ND, Cheng B, Kemmling a, Thomalla G, Fiehler J. ANTONIA Perfusion and Stroke. A Software Tool for the Multi-purpose Analysis of MR Perfusion-weighted Datasets and Quantitative Ischemic Stroke Assessment. Methods Inf Med. 2014;53: 1–13. 25301390 10.3414/ME14-01-0007

[16] Herve D, Mangin J-F, Molko N, Bousser M-G, Chabriat H. Shape and Volume of Lacunar Infarcts: A 3D MRI Study in Cerebral Autosomal Dominant Arteriopathy With Subcortical Infarcts and Leukoencephalopathy. Stroke. Lippincott Williams & Wilkins; 2005;36: 2384–2388. doi: 10.1161/01.STR.0000185678.26296.38 16224090 10.1161/01.STR.0000185678.26296.38

[17] Asdaghi N, Pearce LA, Nakajima M, Field TS, Bazan C, Cermeno F, et al Clinical correlates of infarct shape and volume in lacunar strokes: The secondary prevention of small subcortical strokes trial. Stroke. 2014;45: 2952–2958. doi: 10.1161/STROKEAHA.114.005211 25190442 10.1161/STROKEAHA.114.005211 PMC4198938

[18] Ogata T, Nagakane Y, Christensen S, Ma H, Campbell BC V, Churilov L, et al A topographic study of the evolution of the MR DWI/PWI mismatch pattern and its clinical impact: A study by the EPITHET and DEFUSE investigators. Stroke. 2011;42: 1596–1601. doi: 10.1161/STROKEAHA.110.609016 21512174 10.1161/STROKEAHA.110.609016

[19] Olivot J-M, Mlynash M, Thijs VN, Purushotham A, Kemp S, Lansberg MG, et al Geography, structure, and evolution of diffusion and perfusion lesions in Diffusion and perfusion imaging Evaluation For Understanding Stroke Evolution (DEFUSE). Stroke. NIH Public Access; 2009;40: 3245–51. doi: 10.1161/STROKEAHA.109.558635 19679845 10.1161/STROKEAHA.109.558635 PMC2753724

[20] Wardlaw JM, Smith C, Dichgans M. Mechanisms of sporadic cerebral small vessel disease: Insights from neuroimaging. The Lancet Neurology. 2013 pp. 483–497. doi: 10.1016/S1474-4422(13)70060-7 23602162 10.1016/S1474-4422(13)70060-7 PMC3836247

[21] Hart RG, Diener H-C, Coutts SB, Easton JD, Granger CB, O’Donnell MJ, et al Embolic strokes of undetermined source: the case for a new clinical construct. Lancet Neurol. Elsevier; 2014;13: 429–438. doi: 10.1016/S1474-4422(13)70310-7 10.1016/S1474-4422(13)70310-7 24646875

[22] Jorgensen HS, Nakayama H, Reith J, Raaschou HO, Olsen TS. Acute Stroke With Atrial Fibrillation: The Copenhagen Stroke Study. Stroke. 1996;27: 1765–1769. doi: 10.1161/01.STR.27.10.1765 8841326 10.1161/01.str.27.10.1765

[23] Bhatt A, Majid A, Razak A, Kassab M, Hussain S, Safdar A. Predictors of occult paroxysmal atrial fibrillation in cryptogenic strokes detected by long-term noninvasive cardiac monitoring. Stroke Res Treat. 2011;2011: 172074 doi: 10.4061/2011/172074 21423555 10.4061/2011/172074 PMC3056431

[24] Alhadramy O, Jeerakathil TJ, Majumdar SR, Najjar E, Choy J, Saqqur M. Prevalence and predictors of paroxysmal atrial fibrillation on holter monitor in patients with stroke or transient ischemic attack. Stroke. 2010;41: 2596–600. doi: 10.1161/STROKEAHA.109.570382 20947860 10.1161/STROKEAHA.109.570382

[25] Kim YD, Hong HJ, Cha MJ, Nam CM, Nam HS, Heo JH. Determinants of infarction patterns in cardioembolic stroke. Eur Neurol. 2011;66: 145–150. doi: 10.1159/000330563 21876359 10.1159/000330563

[26] Depuydt S, Sarov M, Vandendries C, Guedj T, Cauquil C, Assayag P, et al Significance of acute multiple infarcts in multiple cerebral circulations on initial diffusion weighted imaging in stroke patients. J Neurol Sci. Elsevier B.V.; 2014;337: 151–155. doi: 10.1016/j.jns.2013.11.039 24332593 10.1016/j.jns.2013.11.039

[27] Gan R, Sacco RL, Kargman DE, Roberts JK, Boden-Albala B, Gu Q. Testing the validity of the lacunar hypothesis: the Northern Manhattan Stroke Study experience. Neurology. 1997;48: 1204–1211. doi: 10.1212/WNL.48.5.1204 9153444 10.1212/wnl.48.5.1204

[28] Kamel H, Okin PM, Elkind MS V, Iadecola C. Atrial Fibrillation and Mechanisms of Stroke: Time for a New Model. Stroke. 2016;47: 895–900. doi: 10.1161/STROKEAHA.115.012004 26786114 10.1161/STROKEAHA.115.012004 PMC4766055

[29] Kolominsky-rabas PL, Weber M, Gefeller O, Neundoerfer B, Heuschmann PU. Epidemiology of Ischemic Stroke Subtypes. 2001; 2735–2740. 10.1161/hs1201.100209 11739965

[30] Lee BI, Nam HS, Heo JH, Kim DI. Yonsei Stroke Registry. Cerebrovasc Dis. 2001;12: 145–151. doi: 10.1159/000047697 11641577 10.1159/000047697

[31] Ay H, Arsava EM, Andsberg G, Benner T, Brown RD, Chapman SN, et al Pathogenic ischemic stroke phenotypes in the NINDS-stroke genetics network. Stroke. 2014;45: 3589–3596. doi: 10.1161/STROKEAHA.114.007362 25378430 10.1161/STROKEAHA.114.007362 PMC4286169

[32] Aerts HJ, Velazquez ER, Leijenaar RT, Parmar C, Grossmann P, Carvalho S, et al Decoding tumour phenotype by noninvasive imaging using a quantitative radiomics approach. Nat Commun. 2014;5: 4006 doi: 10.1038/ncomms5006 24892406 10.1038/ncomms5006 PMC4059926

